# Case not closed: the mystery of the origin of the carpel

**DOI:** 10.1186/s13227-021-00184-z

**Published:** 2021-12-15

**Authors:** Beatriz Gonçalves

**Affiliations:** School of Biological Sciences, University of Bristol, Bristol, UK

**Keywords:** Carpel, Flowering plants, Angiosperms, Plant evolution, Morphogenesis, Patterning, Laminar growth

## Abstract

The carpel is a fascinating structure that plays a critical role in flowering plant reproduction and contributed greatly to the evolutionary success and diversification of flowering plants. The remarkable feature of the carpel is that it is a closed structure that envelopes the ovules and after fertilization develops into the fruit which protects, helps disperse, and supports seed development into a new plant. Nearly all plant-based foods are either derived from a flowering plant or are a direct product of the carpel. Given its importance it’s no surprise that plant and evolutionary biologists have been trying to explain the origin of the carpel for a long time. Before carpel evolution seeds were produced on open leaf-like structures that are exposed to the environment. When the carpel evolved in the stem lineage of flowering plants, seeds became protected within its closed structure. The evolutionary transition from that open precursor to the closed carpel remains one of the greatest mysteries of plant evolution. In recent years, we have begun to complete a picture of what the first carpels might have looked like. On the other hand, there are still many gaps in our understanding of what the precursor of the carpel looked like and what changes to its developmental mechanisms allowed for this evolutionary transition. This review aims to present an overview of existing theories of carpel evolution with a particular emphasis on those that account for the structures that preceded the carpel and/or present testable developmental hypotheses. In the second part insights from the development and evolution of diverse plant organs are gathered to build a developmental hypothesis for the evolutionary transition from a hypothesized laminar open structure to the closed structure of the carpel.

## Introduction

**Fig. 1:**
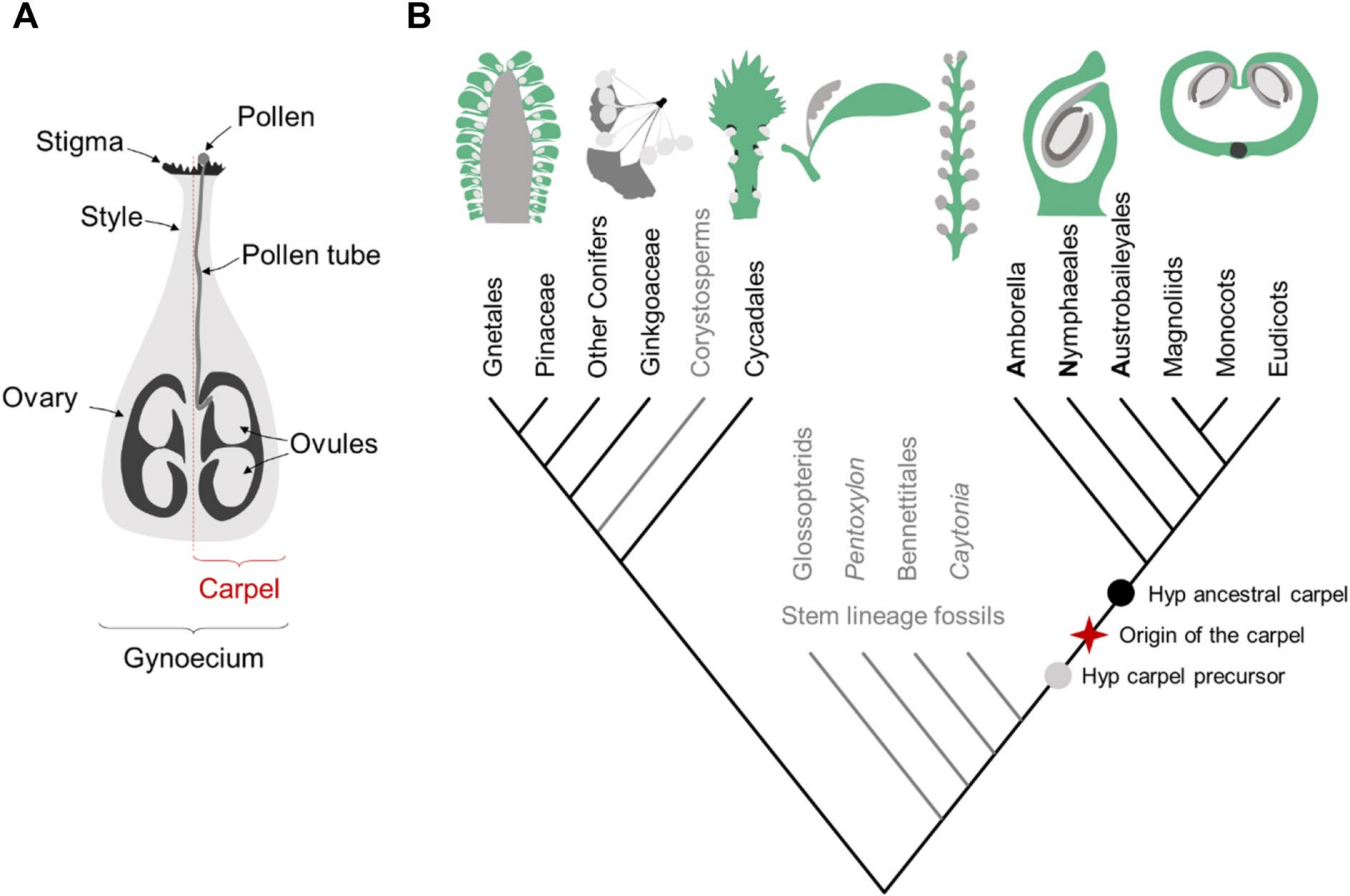
Schematic representation of the carpel and phylogenetic relationships of seed plants. **A** The carpel consists of an ovary which produces the ovules, the stigma which receives the pollen and the style which guides the pollen tube to the ovules. One or more carpels can exist per flower and are assembled in the gynoecium. **B** Relationships within the crown groups of Angiosperms and Gymnosperms and the position of fossil seed plants (in grey writing) are based on Doyle [7]. The evolutionary transition from the precursor of the carpel to the ancestral carpel, marked as “origin of the carpel”, remains difficult to describe and accurately place in time. Two approaches have been prescribed to help pinpoint it: in a “top-down” approach reconstruction of character states in the ancestral carpel is achieved based on character states in extant angiosperms, particularly the early diverging lineages of the ANA grade (this hypothetical ancestral carpel sits somewhere between the origin of the carpel and the root of the crown angiosperms, represented with a black dot); in a “bottom-up” approach character states in the ancestral, or precursor of the carpel are reconstructed based on the closest known relatives of the angiosperms (this hypothetical carpel precursor sits somewhere between the closest known fossil relatives of angiosperms and the time point of the origin of the carpel, represented with a grey dot) [10]. Representative morphologies of female reproductive structures are show above the tree with the different tissues that have been hypothesized as homologs or precursors to the carpel in green. From left to right: conifer cone in longitudinal section, *Ginkgo* reproductive shoot, *Cycas* megasporophyll, *Glossopteris* cupule-bract unit, *Caytonia* cupules on reproductive axis, ascidiate carpel in longitudinal section, plicate carpel in transverse section

The carpel is a complex closed structure that produces the ovules and facilitates fertilization of the egg cells within, via three specialized structures. The stigma receives the pollen, and the style guides the pollen tube and sperm cells towards the ovary, where ovules are contained (Fig. 1A). After fertilization, as the ovule develops into the seed, the carpel tissues develop into the fruit layers which protect and help disperse the seed. The origin of the carpel and the associated tissues that produce and anchor the ovules, facilitate fertilization, protect the seed, and promote seed dispersion, contributed greatly to making flowering plants the most diverse and evolutionarily successful plant lineage. The presence of the carpel is the unifying trait of all flowering plants and gives meaning to the name of the flowering plants group, the angiosperms, which is to say encased or enclosed seed. In contrast gymnosperms, the closest extant relatives of angiosperms, have as the name suggests naked seeds that remain exposed after fertilization. Gymnosperm ovules appear on scales or at the tip of reproductive axes, protected only by their integument and occasionally by bracts or arils, and are fertilized via a pollination droplet. We can consider these gymnosperm reproductive organs as *open* structures as opposed to the *closed* structure of the carpel which encases the seed.

Given the importance of the carpel in the evolutionary history of flowering plants and our own agronomic efforts, its origin has justifiably been the focus of much interest and research on the part of plant and evolutionary biologists. From an evolutionary developmental point of view understanding the origin of the carpel encompasses deciphering both the developmental mechanisms that produced its open precursor and how those mechanisms could have been modified to produce its novel, closed structure. The origin of the carpel, therefore, can be divided into two questions: what did the original carpel look like; and what did the structure that preceded it look like? Below we’ll look at different attempts to answer these questions, current working hypotheses, and what new research directions can take the field further.

### What did the original carpel look like?

**Fig. 2:**
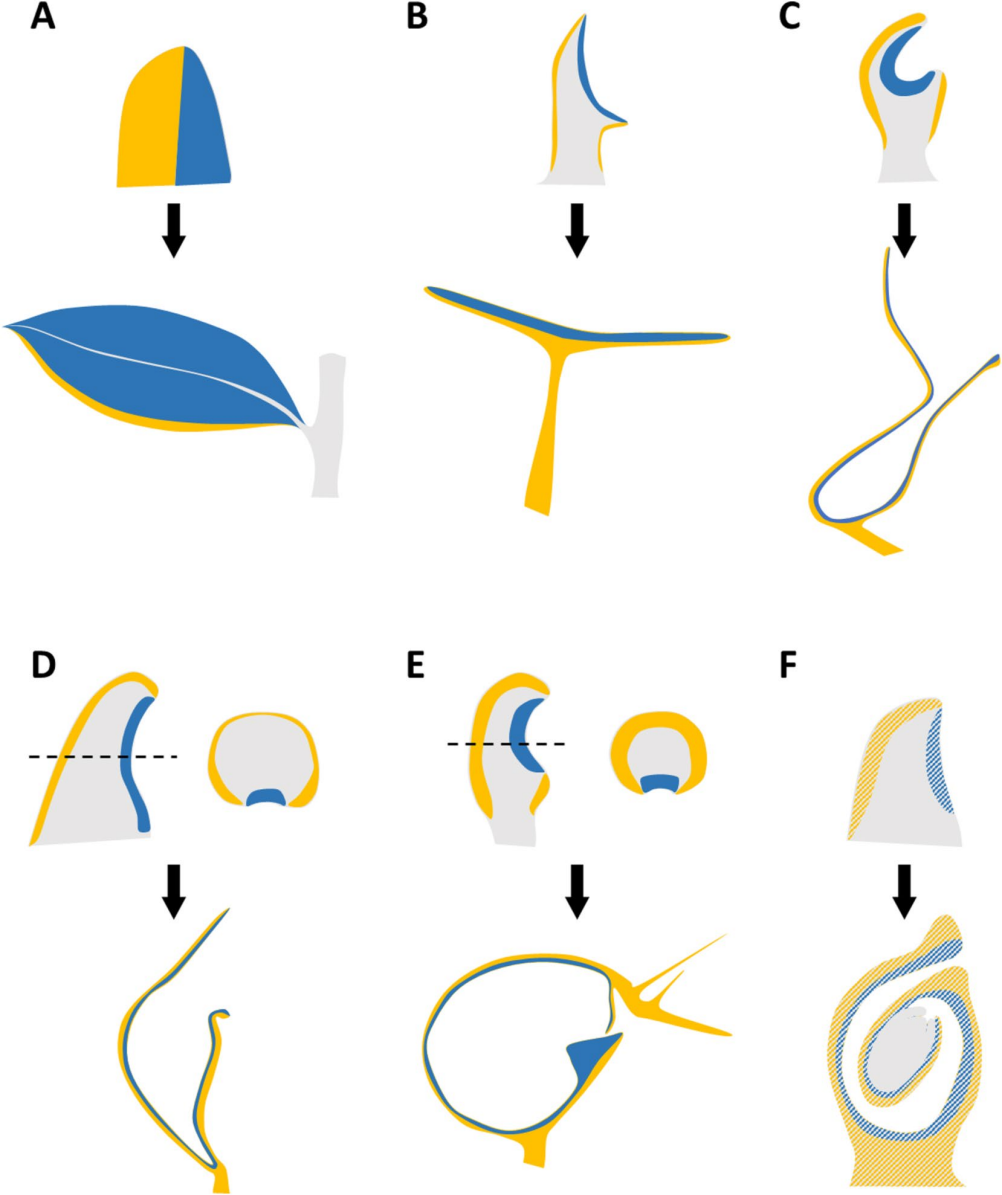
Lateral organ laminar growth diversity and adaxial–abaxial patterning. Primordia and mature shapes are shown in longitudinal midline sections (expect for the bifacial leaf) and the known and hypothesized adaxial and abaxial domains are depicted in blue and yellow, respectively. **A** Bifacial laminar leaf. **B** Peltate leaf. **C** Elaborate peltate petal. **D** Epiascidiate leaf-like Sarracenia traps. **E** Epiascidiate leaf-like Utricularia traps. **F** Ancestral ascidiate carpel structure, reconstructed based on the carpels found in the species of early diverging angiosperm lineages (based on Doyle 2008) [15]. Adaxial and abaxial domains are shaded to reflect a hypothetical unconfirmed distribution

The first question has been addressed with increasing success by a series of character reconstruction studies that combine an ever-growing number of morphological and molecular data from fossil and extant species. The latest and most comprehensive study [1] concluded that the ancestral angiosperm flower had multiple free carpels which were simple, with distal openings closed by secretion. This result agrees with previous studies by Endress and Doyle [2, 3] who have built a wealth of knowledge on the morphology of flowering plants and the reproductive organs of early diverging angiosperm lineages. According to these studies the ancestral carpel was likely of the ascidiate type (from the Greek meaning pitcher-shaped) and likely grew like a cup or a hollow tube (Figs. 1B and 2F) [1, 3]. This type of “tubular urn-like structure” is common in the carpels of basal angiosperm lineages [4], but is far less prevalent across the flowering plants than the conduplicate or plicate carpel of mesangiosperms, so called, because it resembles a leaf folded in half (Fig. 1B). The distinction between these two carpel shapes is important because the developmental mechanisms that produce each one (i.e., the molecular and growth patterns that shape them) are likely to differ and, therefore, have an impact on the evo-devo theory of their origin.

The prevalence of plicate or folded carpels in angiosperms inspired many early theories of carpel evolution. In these early views the carpel was described as a modified leaf and its mode of evolution as the transformation of ovule-bearing leaves by a curling-in or folding-in process [5]. Fusion along the modified leaf margins would complete the evolutionary transition into a closed structure with the ovules on the inside. This folding process, which is tightly linked to observations of the development of folded carpels, was reasonable to assume before the phylogenetic tree of angiosperms and the identity of its basal lineages was resolved. The current angiosperm phylogeny, however, places Amborella as sister-species to all other extant angiosperms, followed by the Nymphaeales and the Austrobaileyales (Fig. 1B) [6]. Members of this group, called the ANA-grade, have ascidiate carpels [4] which supports the hypothesis that the ancestral carpel was ascidiate, with the plicate carpel being a subsequent modification [7]. As it becomes clear that the folded-leaf theory of carpel origin does not fit the more likely scenario that the ancestral carpel was cup-shaped rather than folded, it is perhaps time to review the conduplicate carpel narrative which still colours literature views on carpel evolution and development [8, 9].

### What did the structure that preceded the carpel look like?

To understand where the carpel comes from, we need to reconstruct the character states of reproductive structures in the stem lineage of angiosperms immediately before the evolutionary transition that led to the origin of the carpel (Fig. 1B). To reconstruct the structure that preceded the carpel (also referred to as the ancestor of the carpel, not to be confused with the ancestral or original carpel) it is useful to know at which point in the stem lineage of the angiosperms that structure appeared and what the closest relatives to that point are. There are several reasons why these questions remain some of the biggest mysteries of plant evolution, namely, the great morphological gap between the carpel and the reproductive structures of their closest living relatives [11, 12]. In addition, important gaps in the fossil record of early angiosperms means the relationships within its stem lineage and between extant angiosperms and extant gymnosperms remain inconclusive [13, 14].

Of these unanswered questions, which fossil group is the sister-species to the flowering plants has the greatest impact on a modern developmental theory of carpel origin. The fossil species *Caytonia* is considered by some to be the best candidate for the sister-group to all flowering plants, followed by the extinct Bennetitales and the glossopterids [7]. Regardless of the order in which these groups stand on the stem lineage of angiosperms their reproductive structures are the current best references for the ancestor of the carpel. *Caytonia*’s ovules are enclosed within fleshy cupules that are produced along an axis also described as a rachis (Fig. 1B) [15]. These cupules are described as laminar structures and the ovules are placed on the adaxial surface. Glossopterids are also described as having laminar cupules, but these are attached to the midrib of a leaf, or bract, that is born on an axillary branch (Fig. 1B). While these cupules were initially hypothesized as homologous structures to the carpel they are now more commonly accepted as precursors to the outer integument of angiosperm ovules [14, 16]. According to that hypothesis both *Caytonia* and glossopterid cupules could be transformed into bitegmic ovules by a reduction of the number of ovules to one which is considered the ancestral state in angiosperms [3]. Where the implications of the position of *Caytonia* and glossopterids in relation to angiosperms differ is on the structure that could have given rise to the carpel. In the scenario where glossopterids represent the precursor state, the outer wall of the carpel could be derived from the axillary leaf/bract covering the cupule on the abaxial side and an adaxial cross zone outgrowth derived from the axillary branch. On the other hand, if *Caytonia*’s reproductive structure represents the precursor state, the origin of the carpel would require expansion of the rachis to cover the cupules [15].

Although these theories of carpel origin rest mostly on morphological comparison of reproductive structures, they could be built upon to provide developmental and genetic hypotheses of the evolutionary transition that gave rise to the carpel. A key element of reproductive morphology which can support or undermine the likelihood of a structure being the precursor to carpel, is the identity of the surface on which ovules are produced. From studies in more recently diverging angiosperms lineages, from grasses to *Arabidopsis*, we know that there is a conserved network of genes that control the polarity of lateral plant organs and the identity of tissues on the top and bottom sides of organs, also called the adaxial and abaxial sides, respectively (see more below) [17]. Some of these genes, and/or their relatives, are expressed in carpel tissues with important implications on carpel development and morphology [18]. Ovules of angiosperms are typically produced by tissues that have an adaxial origin or identity [15, 19]. Adaxial and abaxial tissues of plant lateral organs can be recognized by certain key structures, such as the organization of vascular elements. Although we cannot confirm whether the precursor of the carpel expressed adaxial or abaxial genes and whether they determined adaxial and abaxial identity in the female reproductive structures reasonable hypotheses can be put forward based on how these tissues are organized in these species based on anatomical observations of vasculature for example. These could be further supported by studies of how these genes are expressed and what their roles are in other extant seed plants.

### Genetic theories of carpel evolution

Producing a genetic hypothesis for the origin of the flower has been a key goal of plant evolutionary biologists. Most modern theories of carpel origin have been proposed within these larger frameworks for flower evolution [20]. These theories are based not only on morphological comparisons but also gene function conservation between the reproductive structures of flowering plants and what is accepted at the time as their closest living relatives [21]. Consequently, several theories have risen and fallen as the phylogeny of gymnosperms and angiosperms has been revised.

The Mostly-Male (MM) theory was one of the first theories on the origin of the flower to use genetic as well as morphological arguments for the homology between reproductive structures of extant seed plants [22, 23]. It suggests that the architecture of the angiosperm bisexual flower derives mostly from the male reproductive structures of the seed plant ancestor that gave rise to all flowering plants and was formed by the ectopic production of ovules on male sporophylls (leaf-like structures that produce male sporangia). The genetic arguments for this theory are based on the discovery that flowering plants have lost the *NEEDLY* copy of the flower identity gene *LEAFY* (*LFY*), which is involved in specification of female cones in gymnosperms while retaining the *LFY* copy which is involved in the production of male cones in gymnosperms [24, 25]. This theory contrasts with Doyle’s hypotheses in that the carpel is not derived from any structure of the female reproductive units of the flowering plants precursor but from male structures. According to the MM theory the carpel is derived from those microsporophylls that after gaining ovule production, lost the capacity to produce microsporangia. Eventually these sporophylls would have enveloped the ovules to produce the closed carpel. An important assumption of this theory is that the microsporophylls of the ancestor of flowering plants were simple leaf-like structures spirally organized on a stem [22]. This is not consistent with *Caytonia* being the sister-group of angiosperms (if we assume that *Caytonia* shares characteristics with the stem lineage ancestor of the flowering plants), because it does not possess the required laminar structures in the male sporophyll that would have enveloped the carpel [26]. Instead, this theory rests on Corystosperm fossils to fill in the gap for the ancestor of flowering plants. Corystosperms fossils have a central axis on which fertile branches are helically arranged, each producing a seed-bearing cupule. Although some analysis group these and other cupule producing fossils, including *Caytonia,* under the same umbrella of morphological traits [16], most current studies group Corystosperms within gymnosperms, close to Cycadales [7, 11] (Fig. 1B). Another issue with placing Corystosperms as precursors to the flowering plants is that ovules are produced abaxially on these structures which is inconsistent with the adaxial position of ovules on the hypothesized ancestral carpel [15].

Alternative genetic theories for the origin of the flower such as “out-of-male” (OOM) and “out-of-female” (OOF) have also attempted to provide a molecular basis for the transformation of the unisexual reproductive axes of gymnosperms into the bisexual flowers of angiosperms [27, 28]. According to these theories flowers result either from a male cone on which male reproductive structures became restricted to the base and female structures appear at the apex, or from a female cone in which female structures became restricted to the apex and male structures formed at the base. The genetic basis for these transitions rests on a shift in the expression of B-genes during development of the reproductive cone. B-genes are transcription factors that specify floral organ identity within the ABC model of flower development [29, 30]. The ABC model explains floral organ determination according to a modular system, where A function alone specifies sepal identity, A and B together specify petal identity, B and C together specify stamen identity and C alone specifies carpel identity. While B-genes are traditionally responsible for petal and stamen identity in angiosperms they are expressed during male cone development in gymnosperms [31, 32]. According to the “out-of-male” and “out-of-female” theories the bisexual flower could originate either from the exclusion of B-gene expression from the apex of male cones, or from ectopic B-gene expression at the base of female cones. Although this theory does not make an explicit reference to the origin of the carpel, a possible interpretation is that the carpel is homologous to the scales of gymnosperm cones. How the developmental program that generates those scales could have been modified to generate the closed structure of the carpel hasn’t been hypothesized.

The OOM and OOF theories received more attention as new genetic elements of flower development have been discovered. In their version, Baum and Hileman [33] reiterated the stepwise nature of flower evolution, first via the combination of the two sexes into one axis via homeotic conversion of distal microsporophylls into megasporophylls, and later through the compression of the axis, termination of floral meristem and origin of the non-sexual organs. The molecular basis of the homeotic conversion of distal microsporophylls into megasporophylls rests on the accumulation of floral regulator *LFY* along the reproductive axis and the differential expression of B and C genes in response to *LFY* levels. C-genes are responsible for carpel identity and floral meristem identity, while a combination of C and B-genes specifies stamen production [29, 34]. According to Baum and Hileman’s hypothesis intermediate levels of *LFY* at the base of the reproductive axis maintain both B and C expression with concomitant production of male reproductive organs, while higher *LFY* levels in the apex lead to accumulation of C but not B genes and production of female reproductive structures. The discovery of a fourth class of floral organ identity E-genes that form obligatory protein complexes with the products of B and C genes, which are required for reproductive organ identity specification [35] eventually led to a further elaboration of this model. The formation of complexes provided a mechanism through which subtle variations in B and C gene expression levels between the base and the apex of the reproductive axis could lead to different organ specification [36, 37].

The modern theories of flower (and by extension carpel) origin described here intended to reconcile wide morphological gaps with genetic and developmental data, clearly embodying the tenets of evo-devo. Regrettably, neither the MM theory nor the OOM/OOF theories and subsequent elaborations, consider the implications of the structures that gave rise to carpels. Therefore, despite providing a genetic basis for organ identity transformation they do not establish a developmental basis for the transition from one structure shape to the other. Although, the sister-group to angiosperms remains uncertain and we lack a clear picture of what the reproductive structures in the ancestral lineage that gave rise to angiosperms looked like, it is reasonable to hypothesize that the precursor of the carpel would have been an *open* laminar structure bearing a resemblance to a scale as seen in conifers, or a modified leaf (sporophyll) as seen in fossil seed plants. The question then is what was the developmental program that generated those open, laminar, or leaf-like structures, and how was it modified to generate the closed shape of the ancestral ascidiate carpel?

### Revisiting old theories of carpel evolution

The idea that carpels and other floral organs derive from laminar, or leaf-like organs is not an original one. Goethe was perhaps the first to observe the similarities between leaves and floral organs and propose an equivalence between the two [38]. Although he did not explicitly suggest that one gave rise to the other, his ideas grew in popularity and inspired several evolutionary theories on the origin of the flower. Goethe’s comparisons inspired the idea of homology between leaf and floral organs and numerous descriptions of the carpel as modified ovule-bearing leaves. Zimmerman elaborated a unifying theory of plant body organization which provided for the origin of the various plant organs, including leaves and floral organs, through a series of transformations of a basic morphological unit (see Wilson, 1953, for a good overview of the Telome theory and its implications in English, or Zimmerman, 1930, for the original text in German) [39, 40]. According to this theory the carpel would have originated from that simple unit by a process of planation, whereby a branching system is aligned into one plane, followed by webbing or development of tissues between branches to form a lamina, and finally incurvation or folding of the lamina on itself to become closed. This incurvation process is reminiscent of the conduplicate theory of carpel evolution, which was inspired by the striking parallels between leaves and plicate carpels and continues to have a great impact on carpel literature.

While direct comparisons or homology between vegetative and reproductive organs are now avoided, genetic studies showing transformation of leaves into floral organs or the production of floral organs with leaf traits [41–43] as well as conservation of genes across leaf and flower developmental programs [44] have renewed the support for and helped formalize the hypothesis that the evolution of floral organs can be explained by changes to the developmental mechanisms underlying leaf morphology. Although the homology of leaf and floral organs referenced to in flower evolution theories should not be taken too literally, such comparisons may provide a useful framework to understand conservation of gene expression and function between leaf and carpel morphogenesis. Below we’ll look at laminar organ development in plants and how it can inform hypotheses on ancestral carpel development.

### Laminar growth in leaves

Plant lateral organs are shaped in three dimensions by differential growth along three main axes [45, 46]. Growth along the proximodistal axis determines the length of the organ from base to tip. Growth along the mediolateral axis determines width of the organ from a central midline to the margins. In leaves this is called leaf blade expansion. The third axis called the adaxial–abaxial axis, reaches from the top or upper side to the bottom or lower side of the organ and contributes to thickness. Laminar growth is defined by a preferential expansion along two of these dimensions more than the third [47]. Typically, in lateral organs, such as leaves, the adaxial–abaxial dimension remains minimal, while proximo-distal and medio-lateral expansion along the proximo-distal axis produces most of the laminar growth and determine overall leaf-blade shape. Despite its reduced contribution to overall organ growth, patterning of lateral organs along the adaxial–abaxial axis plays a critical role in laminar expansion along the medio-lateral axis and final leaf shape and size [48]. Patterning of the leaf along the adaxial–abaxial axis also establishes an important leaf polarity that is reflected in the formation of specific inner tissues, the organization of vascular tissues, and the differentiation of cell types on the top and bottom of the leaves [49].

Leaf primordia are patterned into adaxial and abaxial domains, that is, respectively, the side adjacent to the meristem and the side that faces away from the meristem, early on during development by two sets of genes, adaxial identity genes and abaxial identity genes [48, 50–54]. The activity of these determinants represses the other identity to create a sharp boundary between the two domains which expresses a third set of genes, boundary genes [55, 56]. A complex network of transcription factors, mobile RNAs and hormone signalling pathways have been found to control leaf patterning along the adaxial–abaxial axis. Among them *HD-ZIP III* family members, *MYB* and *LOB* domain genes specify adaxial identity [48, 50, 57–60], while *KANADI*, *YABBY* and *AUXIN RESPONSE FACTORS* specify abaxial side [54, 61–64]. Known boundary determinants belong to the *WOX* gene family. Establishment of this boundary is necessary for laminar growth along the mediolateral axis, as mutants of either adaxial, abaxial, or boundary identity genes fail to produce laminar growth and either lack leaf-blade expansion or produce entirely radial organs [48, 65]. Occasionally mutants of the adaxial–abaxial patterning module also produce tubular and/or trumpet shaped organs which result from issues with adaxial–abaxial boundary position. These observations led to the suggestion that natural variation in the position of the adaxial–abaxial boundary could account for natural variation in leaf shape and explain extreme examples of leaf shape, such as peltate leaves (see below) [17].

Early experiments on leaf polarity establishment suggested that patterning of the primordium into adaxial and abaxial domains is initiated by a signal from the meristem that specifies adaxial identity [66]. Various adaxial identity determinants have been hypothesized over the time as the identity of the meristem signal, including *HD-ZIP III* genes and small RNA molecules. In addition, a gradient of the hormone auxin across the meristem and the leaf primordium has been implicated in this process as either that signal or a relay of the signal [66, 67]. On the other hand, it has also been proposed that rather than being specified *de-novo* in the primordium, adaxial–abaxial patterning is inherited from a pre-patterning of the meristem itself into an inner zone expressing adaxial determinants, and an outer zone expressing abaxial determinants [65, 68]. Lateral organs emerge, where these two domains meet with an auxin signalling maxima. The auxin signalling pathway feeds back on the adaxial–abaxial patterning module to fine tune the position of the boundary within the primordium [65]. Therefore, a combination of adaxial–abaxial patterning and auxin signalling contributes to laminar growth and ultimately leaf shape determination. Interestingly the pre-patterning of apical meristems was also seen in the inflorescence meristem [65] which could suggest that the adaxial–abaxial module is part of a fundamental process in plant body organization.

### Modifications to laminar growth in other organs

Not all plant lateral organs exhibit the same level of laminar growth. Natural leaf shape diversity can provide great insight into the developmental mechanisms that control morphogenesis and how these can be modified to generate novel forms. Notable variations are the unifacial leaves of some monocots which, unlike the bifacial laminar leaves described above, lack laminar growth of the leaf blade [69]. Unifacial leaves can be radial like the adaxial mutants of the classical bifacial leaves or flattened in the perpendicular orientation to what is common in bifacial leaves. The mechanism for this new growth orientation remains unclear although it appears to be independent of adaxial–abaxial patterning but involve auxin signalling [70]. Interestingly, while these unifacial leaves are completely abaxialized on the distal part as evident from the anatomy of inner tissues, they have a hollow bifacial structure at the base. During formation of this hollow structure at early stages of development the primordia of these unifacial leaves resembles early stages of carpel development, where a longitudinal suture is formed [71, 72]. Another interesting example of altered leaf morphology is that of peltate leaves which have a bifacial lamina that is attached on its abaxial side to a radial petiole (Fig. 2B). This morphology is reminiscent of several adaxial–abaxial mutants which produce trumpet shaped leaves, where the base is entirely radial and the top forms a rudimentary lamina [69]. A study of the peltate leaves of *Tropaeoleum majus* found that this shape is formed by fusion of the leaf margins on the adaxial side of the primordium which produces an adaxial outgrowth called the cross zone and leads to radialization of the petiole [73]. As hypothesized this cross zone was found to express a member of the YABBY family of abaxial determinants suggesting that altered adaxial–abaxial patterning is responsible for this leaf shape variation.

Among lateral organs, petals exhibit some of the most spectacular shape diversity in plants and have been the focus of several developmental studies. This diversity can be seen not just in the extent of blade expansion via laminar growth [74] but through modifications that produce elaborate tridimensional petals and nectar spurs [75]. Elaborate petals in the Ranunculaceae family have a concave structure produced by an adaxial or ventral outgrowth called the upper lip (Fig. 2C). Studies of the *Nigella* genus indicate that this petal shape is a modification of the peltate leaf shape with a radial stalk and a bifacial lamina that forms a cup [76]. Members of genes families involved in specification of the adaxial–abaxial patterning are highly expressed in the early stages of petal development [77]. At a later stage as the ventral outgrowth expands in a laminar fashion it expresses adaxial and abaxial determinants in opposing domains [76]. It is hypothesized that a rearrangement of the adaxial–abaxial patterning is responsible for generating this elaborate shape.

Perhaps the most extreme examples of leaf shape modification are the cup-shaped traps of some carnivorous plants. These complex shapes evolved independently in four separate lineages from ancestors with traditional bifacial leaves. These evolutionary transitions from open laminar structures to near closed ones are reminiscent of the carpel origin. In fact, the traps of carnivorous plans are said to be epiascidiate which shares the same etymology as the ascidiate ancestral carpel (Fig. 2F), both alluding to their cup or vessel like, closed or near closed shape [78]. A long-time interest for the origin of the complex shapes of carnivorous plant traps led to the hypothesis that the inside of their walls is homologous to the top or adaxial side of open or flat leaves, while the outside is homologous to the bottom or abaxial side of leaves. A study of trap development in *Sarracenia purpurea* found that members of adaxial and abaxial gene families are expressed on the outside and inside of the traps, respectively, in a pattern that is coherent with the leaf homology hypothesis [79]. Interestingly rather than having an ectopic domain of abaxial expression on the ventral side like in peltate leaves, adaxial–abaxial gene expression patterns in the trap primordium are modified such that the adaxial determinant is expressed in a narrow longitudinal band on the ventral side, and abaxial determinants occupy an enlarged domain that wraps around from the dorsal to the ventral side of the primordium (Fig. 2D) [79]. In a recent study of cup-shaped leaf development in the traps of *Utricularia gibba* we found a similar expression pattern and showed by functionally overexpressing adaxial gene *UgPHV1* that a restricted adaxial domain is required for trap morphogenesis (Fig. 2E) [47]. Through computational modelling we tested the hypothesis that both the shape and position of the boundary between adaxial and abaxial domains have important consequences for laminar growth and leaf shape.

Together these studies suggest that relatively simple rearrangements of adaxial–abaxial patterning with a restriction of the adaxial domain can underlie dramatic evolutionary transitions from open laminar organs to closed or cup-shaped ones. More importantly it begs the question of whether a similar shift could underlie the transition from the laminar precursor of the carpel to the ascidiate or cup-shaped ancestral carpel. The distribution of adaxial and abaxial domains in the ancestral carpel can be hypothesized based on how these domains occur in the carpels of early diverging angiosperm lineages, which in turn can be inferred by the presence of adaxial and abaxial morphological markers and the expression of adaxial and abaxial identity genes [15, 80, 81]. According to such reconstructions the inner tissues of the carpel have adaxial origin, while the outer tissues are abaxial [12]. Without functional data it is difficult to tell whether the organ patterning function of these genes is conserved in these early diverging lineages. Nevertheless the current framework allows us to move the field forward by proposing testable hypothesis for how changes to the distribution of adaxial and abaxial identity promoters could have led to the production of this novel organ shape. Figure 2F shows one such scenario, where the ascidiate carpel shape is produced by restriction of adaxial identity to a smaller domain of the floral meristem. Other scenarios could involve expansion of the abaxial domain or even an alternative molecular pathway independent of adaxial–abaxial patterning.

### Perspectives on carpel development and evolution

A thorough review of carpel development is beyond the scope of this work and has been written by numerous colleagues in the field who have both synthesized the gene regulatory networks and hormonal signalling pathways that contribute to shaping and tissue differentiation of this organ, and assessed the conservation of these mechanisms in angiosperms and beyond [21, 43, 82–87]. For our current reflection it is important to note that, like other lateral organs, the carpel has a proximo-distal axis, and its walls differentiate into inner and outer surfaces that are considered homologous to the adaxial and abaxial surfaces of leaves, while the mediolateral axis is interpreted as the lateral expansion of carpel walls from a central vascular bundle [19]. Unlike other lateral organs, however, the carpel exhibits a vastly more complex differentiation pattern along each of these axes and its development is likely under the control of unique pathways that may not reflect a common inherited program with leaves. Members of key adaxial and abaxial identity gene families are known to be expressed in the carpel with important consequences to carpel shape and tissue differentiation [18, 54, 61, 64, 87–89]. In addition, intricate patterns of auxin flow and signalling maxima are necessary for proper patterning of growth and specification of domains within the developing carpel [90–92]. Despite this wealth of knowledge, it is not clear how these genes may contribute to early patterning of growth in the primordium such that a closed structure is formed, and how they may have been involved in its evolutionary origin from a structure with laminar open growth. One important difference between carpel and other lateral organs is that the carpel, or carpels, is formed at the centre of the floral meristem and its initiation is concomitant with the termination of meristematic activity. It may very well be that this transition from meristem to carpel formation entrains a wholly different organ patterning process separate from the adaxial–abaxial patterning or lateral organs that occurs at the flanks of the meristem.

The carpel, hidden in plain sight among spectacular floral organs and leaf shapes, is an organ of key importance for the diversification of flowering plants and for our own economy. Its origin is a longstanding mystery that has captured the curiosity of plant and evolutionary biologists alike. With ever more performing imaging methods and the development of new genomics tools we now have nearly unfettered access to the earliest stages of flower and floral organ development [93–95]. On the other hand, there has been a remarkable effort to expand the range of developmental models into the early diverging lineages of flowering plants which will prove invaluable to our understanding of ancestral carpel development [96–101]. Combining these studies with insights from the development and evolution of a range of plant lateral organs could bring a new evo-devo light into the great mystery of carpel origin.

## Data Availability

Not applicable.
